# Spatio-temporal dynamics of a fish predator: Density-dependent and hydrographic effects on Baltic Sea cod population

**DOI:** 10.1371/journal.pone.0172004

**Published:** 2017-02-16

**Authors:** Valerio Bartolino, Huidong Tian, Ulf Bergström, Pekka Jounela, Eero Aro, Christian Dieterich, H. E. Markus Meier, Massimiliano Cardinale, Barbara Bland, Michele Casini

**Affiliations:** 1 Swedish University of Agricultural Sciences, Department of Aquatic Resources, Institute of Marine Research, Lysekil, Sweden; 2 Swedish University of Agricultural Sciences, Department of Aquatic Resources, Institute of Coastal Research, Öregrund, Sweden; 3 Natural Resources Institute Finland (Luke), Turku, Finland; 4 Puolipäivänkatu 4 A 6, Helsinki, Finland; 5 Swedish Meteorological and Hydrological Institute, Research Department, Norrköping, Sweden; 6 Leibniz Institute for Baltic Sea Research Warnemünde, Department of Physical Oceanography and Instrumentation, Rostock, Germany; Technical University of Denmark, DENMARK

## Abstract

Understanding the mechanisms of spatial population dynamics is crucial for the successful management of exploited species and ecosystems. However, the underlying mechanisms of spatial distribution are generally complex due to the concurrent forcing of both density-dependent species interactions and density-independent environmental factors. Despite the high economic value and central ecological importance of cod in the Baltic Sea, the drivers of its spatio-temporal population dynamics have not been analytically investigated so far. In this paper, we used an extensive trawl survey dataset in combination with environmental data to investigate the spatial dynamics of the distribution of the Eastern Baltic cod during the past three decades using Generalized Additive Models. The results showed that adult cod distribution was mainly affected by cod population size, and to a minor degree by small-scale hydrological factors and the extent of suitable reproductive areas. As population size decreases, the cod population concentrates to the southern part of the Baltic Sea, where the preferred more marine environment conditions are encountered. Using the fitted models, we predicted the Baltic cod distribution back to the 1970s and a temporal index of cod spatial occupation was developed. Our study will contribute to the management and conservation of this important resource and of the ecosystem where it occurs, by showing the forces shaping its spatial distribution and therefore the potential response of the population to future exploitation and environmental changes.

## Introduction

Understanding the spatial dynamics of animal populations and using this information in biological conservation and resource management represents one of the new frontiers in marine ecology. Changes in spatial distribution and migration patterns have been reported in aquatic and terrestrial ecosystems, both at small and large spatial scales [1, 2]. These changes have been attributed to climate effects [3–5], biological interactions such as density-dependent responses or predator-prey dynamics [6–9] and human pressures including fishery or hunting [10–13]. Often these causes act simultaneously, and most likely in interaction [14], to trigger large re-location of natural populations.

It is well demonstrated that the abundance and distribution of marine fish populations is affected by both density-dependent (or demographic) and density-independent (or environmental) factors [15–22]. Their effects in isolation or through interactions can also lead to large variations in the spatial distribution of fish populations [23–24]. For instance, expansion of the distribution towards marginal habitats and, at low densities, contraction to the most suitable habitats are known regulatory mechanisms in fish populations to avoid unfavourable conditions, release competition and optimise the use of resources and habitats, as predicted by the ideal free distribution theory [25].

Beside its ecological significance, understanding the processes shaping the spatial distribution of fish populations is crucial for their conservation. In particular, the knowledge of what determines the spatial distribution of exploited populations could provide valid information to regulate the fishery [26] and predict the effects of habitat loss and climate change. Moreover, habitat occupation is a key indicator of species and community status and its quantification is therefore needed for a full implementation of an ecosystem approach to the management of human activities [27].

**Fig 1:**
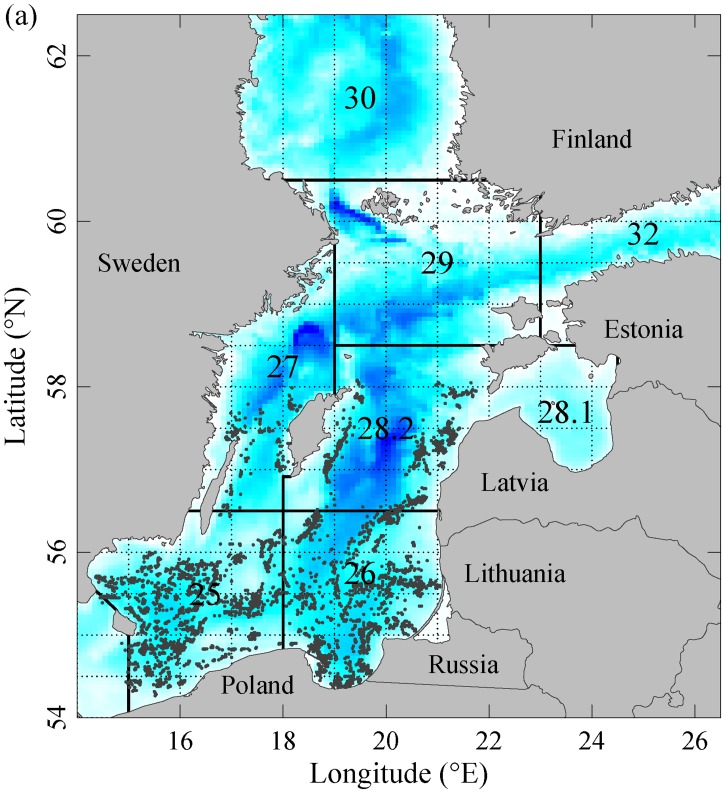
Study area and location of cod trawl hauls. The map (made with Natural Earth) shows ICES Subdivisions (SDs, separated by solid black lines), and the ICES statistical rectangles (thin dotted lines). Bathymetry is shown in shades of blue. The Eastern Baltic cod stock occurs in the area covered by SDs 25–32 (SD 31, covering the Bothnian Bay, is not shown).

**Fig 2:**
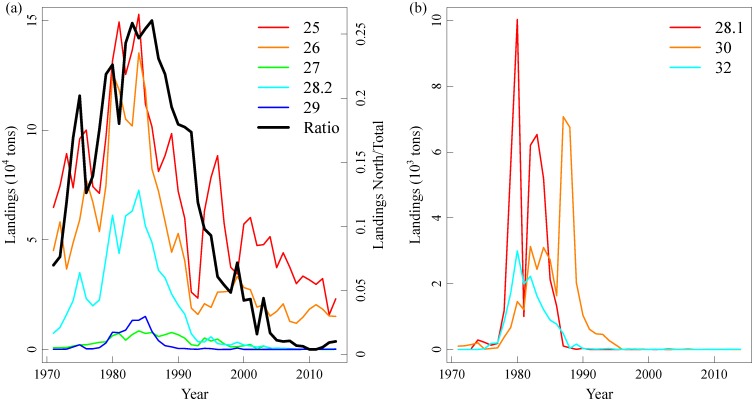
Cod commercial landings. **(a)** Commercial landings in the ICES Subdivisions (SDs) 25–29 (excluding SD 28.1). The black line is the ratio of landings in the northern SDs (SD 27–29) to the total landings (SDs 25–29, excluding SD 28.1). **(b)** Commercial landings in SDs 28.1 (Gulf of Riga), SD 30 (Gulf of Finland) and SD 32 (Bothnian Sea). Landings in SD 31 are not presented because of the very small amount.

The Baltic Sea cod (*Gadus morhua*) is the main piscivorous fish of the open Baltic Sea and, as such, plays a crucial structural and functional role in the Baltic ecosystem [28–30]. During the past three decades, in fact, large variations in the abundance and distribution of the Eastern Baltic cod population (hereafter referred to as Baltic cod, Fig 1) have caused top-down driven multi-level changes in both open sea and coastal food webs [28, 29, 31, 32]. Fisheries statistics show large spatial variations in cod landings over time in the Baltic Sea during the past decades (Fig 2), suggesting that the distribution of the cod population has also varied considerably. Although the causes of temporal variation in the Baltic cod population have been studied in depth [33, 34], no analytical investigation has been performed on the causes of the long-term changes in cod spatial distribution.

Here we used, for the first time, a unique extensive dataset of data collected during international bottom trawl surveys in the last three decades by several countries bordering the Baltic Sea. The aim of our study was to investigate through statistical modelling the effects of density-dependent processes (i.e. mediated by changes in population abundance) and hydrological conditions on the spatio-temporal dynamics of cod in the Baltic Sea. The model adopted was also used to predict the cod population distribution in different periods, and an index of the area occupied was calculated as a novel indicator of the status of the cod population.

## Materials and methods

### Biological and hydrological data

We used cod standardized catch per unit of effort (CPUE, catch in numbers per hour trawling) collected during international trawling surveys by the countries surrounding the Baltic Sea between 1982–2012 (Fig 1, S1 Fig). The surveys have been coordinated as Baltic International Trawl Survey (BITS) by the International Council for the Exploration of the Sea (ICES) since 1998 [35], but a major shift in the sampling gear occurred in 2000 when national trawls were replaced by a common standard TV-3 trawl used by all countries. Conversion factors among national and TV-3 trawl types were estimated to standardise CPUE by application of a machine learning algorithm based on generalized regression neural networks [36]. The algorithm was trained on the 2000–2009 dataset and tested on the 1998–1999 dataset to account for year, month, country, geographical position, and depth related differences in trawls’ catching efficiencies by cod age group (see S1 Text for more details on trawl standardisation). Each record contains the haul location information (latitude and longitude), trawling depth and the CPUE of cod by age-class (ages 1 to 10). We constrained our analyses to the period 1982–2009 because of the lack of fine-scale hydrological data after this year (see below). We focused on the adult component of the cod population (ages 3 and above) sampled between January to March. The surveys cover the major area of occurrence of the Eastern Baltic cod population, i.e. the ICES Subdivisions (SDs) 25–28 (Fig 1). The time series of cod population size was from the latest accepted analytical stock assessment [37].

All the cod CPUE observations in time and space were matched to modelled hydrological data. Monthly mean oxygen and salinity data were interpolated from model results of the Swedish Coastal and Ocean Biogeochemical model coupled to the Rossby Centre Ocean circulation model (RCO-SCOBI) [38, 39, S2 and S3 Figs]. RCO-SCOBI is a three-dimensional model of the Baltic Sea with a horizontal grid resolution of about 3.7 km (2 nautical miles) and with 83 vertical levels with layer thicknesses of 3 m. The physical and biogeochemical sub-models describe the dynamics of water temperature, salinity, currents, sea level, sea ice, but also nitrate, ammonium, phosphate, phytoplankton, zooplankton, detritus and oxygen. RCO-SCOBI is forced with three-hourly atmospheric data from a regionalized re-analysis simulation [40] and with monthly river runoff and nutrient load data [39] for the period 1961–2007. In earlier studies the model was successfully used to study past climate variability (e.g. see [41]) and future projections [42]. The original model output was aggregated on the coarser ICES rectangles, but the vertical resolution of 3 meters remained unchanged. Each cod CPUE observation was matched to the average hydrological conditions within the same ICES rectangle, at the depth and month of trawling. We used salinity and oxygen because of their recognized importance as driver of the Baltic Sea biota, especially in relation to cod [43]. We used a relatively coarser spatial resolution of hydrological conditions compared to the horizontal grid resolution of the hydrological model because model results were only validated at the sub-basin scale due to the lack of observations at high resolution below the sea surface. In addition, no assimilation of observations into the model was applied and the chaotic behavior of the ocean’s mesoscale does not allow a one-to-one comparison with observations in any case. To estimate how representative the hydrological parameters salinity and oxygen averaged within each ICES rectangle are for all the locations of the model grid within each ICES rectangle, we calculated the spatial variance at each depth for different rectangles that vary between 2 times 2 and 15 times 15 model grid boxes. The spatial variances were calculated every second day and then averaged for a 30-year period between 1966 and 1995. As expected, we found a moderate increase of the spatial variance averaged for the entire Baltic Sea with the size of the rectangles but also an approximate saturation of variances for rectangles larger than about 8 times 8 model grid boxes. Moreover, spatial variances at all spatial scales within ICES rectangles seem to be much smaller than temporal variances at seasonal to decadal time scales. Hence, we assume that there is no real added value for the modeling of cod distributions by using information from hydrological parameters at the high horizontal resolution of the model grid. Further investigations of the impact of spatial variability at various scales on cod distributions are out of the scope of the present publication.

To investigate the potential relation between cod distribution and spatio-temporal variations in recruitment, we used time-series of the cod reproductive volume by SD. The reproductive volume (RV) is defined as the volume of water with a salinity > 11 psu and an oxygen concentration > 2 mL L^-1^, which are recognized as suitable hydrographic conditions for the development of cod eggs [44]. The reproductive volume is acknowledged to be a key driver of cod recruitment success in the Baltic Sea [45]. In this study RV was used as predictor of cod CPUE in each SD, with the hypothesis that cod adults would tend to remain close to the areas where they were born, i.e. have a higher probability to stay in the same SD than to spread into other SDs. This accounted for the hypothesis that the spatial expansion/contractions of the cod population are driven by changes in the location of suitable spawning areas [46].

### Statistical modeling

In our model, three spatial scales are included, tackling different mechanisms potentially capable to shape the distribution of the adult fish: the small scale (hydrological conditions by ICES rectangle) indicates the habitat preferences/requirements of fish, the basin scale (reproductive volume by SD) investigates the effect of the extent and location of suitable spawning area on the future distribution of the adults born in those areas, while the regional scale (total population size in the Baltic Sea) analyses the density-dependent effect investigating the demographic effect of population size on the fish distribution over the whole central Baltic Sea.

Regression methods are the main approaches to analyze the relationships between species abundance (or other response variables) and their environment [47]. Considering the versatility and flexibility in modeling the effects of density-dependent and density-independent variables [5, 17, 20, 22, 48], we used Generalized Additive Models (GAMs) [49, 50]. GAMs assume that the response variable follows some specific statistical distribution, such as Gaussian, Poisson, etc. However, biological data often violate this assumption, and issues like zero-inflation (more zeroes than the specified distribution suggests) and over-dispersion (variance exceeds the mean) are frequently encountered [51–53]. In our case, most of the trawl data were collected during years of low cod population size and for this reason, we used the quasi-Poisson distribution, which gives higher weights to larger CPUEs and at the same time is able to handle zero-inflation. The variance of a variable Y that follows a quasi-Poisson distribution should be a linear function of the mean (expected value) of Y: Var(Y) = θ*E(Y), where *θ* (> 1) is the dispersion parameter [54].

The choice of predictors used in this study was based on information from official fisheries landings and acknowledged ecological processes driving cod dynamics (see also Biological and hydrological data, above). Landing statistics have shown that when cod population size was high, as in the late 1970s to the early 1980, large amounts of cod were landed in the northern areas of the Baltic Sea. On the contrary, during periods of low population size, most of the landings were from the southern part of the Baltic Sea (Fig 2). This suggests a geographical expansion and contraction of the population at high respective low population sizes. Therefore, we used population size from stock assessment estimates [37] as density-dependent predictor of cod CPUEs. Salinity and oxygen are the major hydrological factors known to influence cod abundance and distribution [55], with high values being physiologically favorable for this marine species [43]. Moreover, variations in cod reproductive volume (RV) have been acknowledged as major driving forces of cod recruitment [44]. Trawl haul depth was also included in the models in accordance to previous studies showing the preference of cod for certain depths [55]. To test different hypotheses of density-dependence and density-independence, we built the following models investigating the spatio-temporal dynamics of the adult component of the cod population (CPUE at ages 3+):

mod1: f(CPUE)=α+s1(lon, lat)+s2(lon, lat)⋅population+s3(oxy, sal)+s4(depth)+s5(RV)+ε

mod2: f(CPUE)=α+s1(lon, lat)+β⋅(population)+s3(oxy, sal)+s4(depth)+s5(RV)+ε

mod3: f(CPUE)=α+s1(lon, lat)+s2(lon, lat)⋅population+s3(depth)+s4(RV)+ε

mod4: f(CPUE)=α+s1(lon, lat)+s2(lon, lat)⋅population+s3(oxy, sal)+s4(depth)+ε

mod5: f(CPUE)=α+s1(lon, lat)+s2(lon, lat)⋅population+s3(depth)+ε

mod6: f(CPUE)=α+s1(lon, lat)+s2(oxy, sal)+s3(depth)+ε

mod7: f(CPUE)=α+s1(lon, lat)+s2(oxy)+s3(sal)+s4(depth)+ ε

where *f* is the log-link function for quasi-Poisson distribution, *α* the model intercept, *β* the parametric coefficient and *s*_*1*_*-s*_*5*_ the smoothing functions with no prior constraint on the maximum number of knots. *lon*, *lat*, *oxy*, *sal* and *depth* are the longitude, latitude, oxygen, salinity and depth at each sampling location, respectively, *RV* is the reproductive volume by SD (lagged 3 years back to match with the most abundant age-class included in the adult CPUE, i.e. age 3) and *population* the total cod population size (ages 3+) at a specific year. *ε* is the error term whose variance should have, in the case of quasi-Poisson distribution, a positive linear relationship with CPUE.

The hypothesis of density-dependency is tested with the two alternative formulations presented by model.1 and model.2 where the linear effect of cod population size is assumed to be spatially-variable by the term *s(lon*, *lat) ·population* [20, 22] and spatially-invariant by the term *β·population*, respectively. Models.3-5 test for the inclusion of the different environmental variables into models with spatial density-dependence. Model.6 and model.7 assume that only density-independent environmental variables affect cod spatial dynamics and they are used to test for the interacting and additive effects of *oxygen* and *salinity*, respectively. All models assume that the response variable follows a quasi-Poisson distribution, which means the variance of the model residuals should have a linear relationship with the fitted values. Model selection was based on the analysis of deviance explained and minimization of the generalized cross validation (GCV, [56]). In addition, the predictive power of each model was compared using a bootstrap approach based on the genuine cross validation score (gCV). Each model was fitted on 80% of the data, randomly selected, and the average squared prediction error was calculated on the data points removed. This procedure was repeated 1000 times for each model and the gCV was calculated as the mean of this error statistic [57]. The best model was checked for violation from the main model assumptions including spatial auto-correlations in the model residuals (S5 Fig). All analyses were conducted in R (www.r-project.org) using the mgcv library [50].

## Results

**Fig 3:**
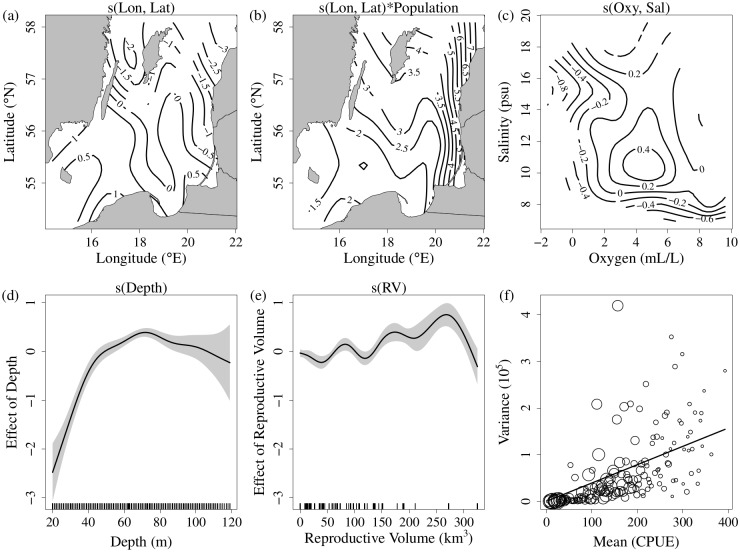
Results of the best model. **(a)** effect of spatial location. **(b)** spatial effect of cod population size. **(c)** interactive effects of salinity and oxygen. **(d)** effect of depth. **(e)** effect of the reproductive volume. **(f)** estimated variance-to-mean relationship (solid line, slope equals to 268). The circles are averaged squared residuals in each category (e.g. 0 < *E(Y) < 2*, 2 < *E(Y) <* 4 and so on).

The model selection procedure showed that model.1 performed better than the other models, indicating that cod spatio-temporal dynamics during the past thirty years were driven by both spatial density-dependence and hydrographic conditions (Table 1). The combination of both better statistics in comparison to model.2 and estimation of a statistically significant spatially-variable effect of population size (Fig 3b) support the hypothesis that a spatially heterogeneous density-dependent process contribute to explain cod CPUE. Moreover, all the model statistics considered favour model.6 in comparison to model.7 suggesting an interacting rather than additive effect of oxygen and salinity on the cod local densities.

**Table 1:** Model selection results. Intercept (A), linear coefficient (β)and estimated degrees of freedom of smoothing terms are shown for all the models. The deviance explained (dev.expl), the generalized cross validation (GCV) and genuine cross validation (gCV) scores are also indicated for each model. All the terms of each model are significant (p-value < 0.001).

Model	Predictors								dev.expl	GCV	gCV
	A	s(lon,lat)	s(lon, lat) · population	β·(population)	s(oxy, sal)	s(oxy) + s(sal)	s(depth)	s(RV)			
Model.1	4.2	26.1	18.4		21.9		6.3	8.5	44.0	252.6	356.1
Model.2	4.3	28.1		2.4	25.4		6.1	8.8	41.0	264.5	362.3
Model.3	4.2	26.1	21.2				6.2	8.5	41.7	260.4	359.0
Model.4	4.3	26.1	18.6		21.2		6.3		42.7	257.2	357.7
Model.5	4.2	26.0	21.1				6.2		40.6	264.1	359.0
Model.6	5.0	28.0			26.8		6.3		27.3	324.7	390.9
Model.7	5.0	28.2				6.5; 8.9	6.6		25.3	331.5	394.0

The results of model.1 (best model) are shown in Fig 3. The term *s(lon*, *lat)* shows the isolated effect of spatial location on cod CPUE. High CPUEs appeared in the south (especially SD 25) and low CPUEs in the north of the study area (Fig 3a). The effect of population size has a pronounced positive spatial gradient towards the northeast of the study area. In practice, when the cod population size decreases, the local CPUEs in the northeastern decrease more than in the rest of the Baltic Sea. Accordingly, local CPUEs tend to increase more in the northeastern part of the distribution when the cod population size increases (Fig 3b). The interaction term between oxygen and salinity shows that when the salinity is low, the effect of oxygen is weak, and salinity is the limiting factor; conversely, when the concentration of oxygen is low, the effect of salinity is weak, and oxygen is the limiting factor (Fig 3c). Depth shows a clear non-linear effect on cod CPUE: the effect increased rapidly until around 50 meters, peaked at around 70 meters, and slightly decreased thereafter (Fig 3d). The effect of the reproductive volume (RV) is slightly confounded by a tendency to overfit the data, but sensitivity analysis on the level of smoothing confirms the positive relationship with the cod CPUE three years later (Fig 3e). The effect drops for RV > 270 km^3^ but this is driven by the last high value of RV. By removing one covariate at a time from the best model and calculating the relative decrease in the deviance explained, we evaluated the contribution of each variable to explain the local CPUE of cod in the best model (S1 Table). The spatial density-dependent term is by far the most relevant factor with a drop of almost 32% in the deviance explained by the model excluding this term. The second and third terms by relevance are the geographical coordinates and depth with a contribution to the variance of only 8% and 7% respectively. The terms that included the hydrographic variables appear as the less influential with a contribution of 5% or less.

The estimated dispersion parameter (*θ*) = 268 suggests that solid overdispersion exists in our data. We also plotted averaged squared residuals and fitted values for categories 0 < *E(Y) < 2*, *2* < *E(Y) < 4* and so on, to diagnose the linear relationship between variance and mean, typical of a quasi-Poisson distribution (Fig 3f). The diameters of the circles are proportional to the number of samples for each category, and all categories have at least 10 samples. The variogram of the residuals showed no spatial auto-correlation in the residuals of the best model (data not shown).

**Fig 4:**
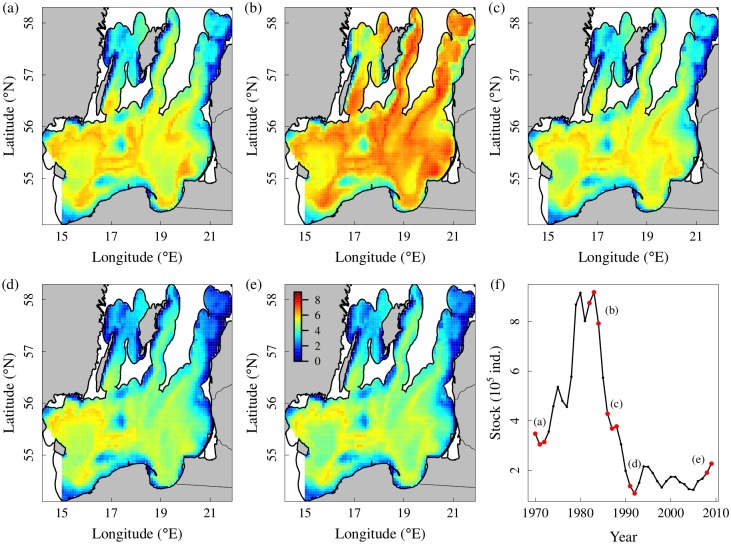
Predictions of cod spatio-temporal population distribution at different population levels. **(a)** 1970–1972 (hindcast), **(b)** 1982–1984, **(c)** 1986–1988, **(d)** 1991–1992, **(e)** 2006–2007. **(f)** Baltic cod population size (ages 3+). Red and blue colors indicate high and low predicted CPUEs, respectively.

Using the fitted best model (model.1), we predicted cod distribution for five different periods characteristic for the population dynamics observed in the last 30 years (Fig 4). In the early 1980s, during a period of high population size (Fig 4b), our model predicted generally higher local densities and a wider spatial distribution that extended across most of the study area. In the mid 1980s the population size had an abrupt decrease to levels comparable with those observed in the early 1970s (Fig 4a and 4c), and the model predicted an overall reduction of local densities, but with a major decrease observed in the northeast (i.e. SD 28). In the early 1990s (Fig 4d) the cod population size reached a historical minimum, and the model predicted a further contraction in the distribution mainly towards the southwestern part of the study area (i.e. SD 25). During the most recent years (Fig 4e), a moderate increase in the population size produced minor expansion of the distribution towards the southeast (i.e. SD 26), but not in the northern part of the study area (i.e. SDs 27 and 28).

**Fig 5:**
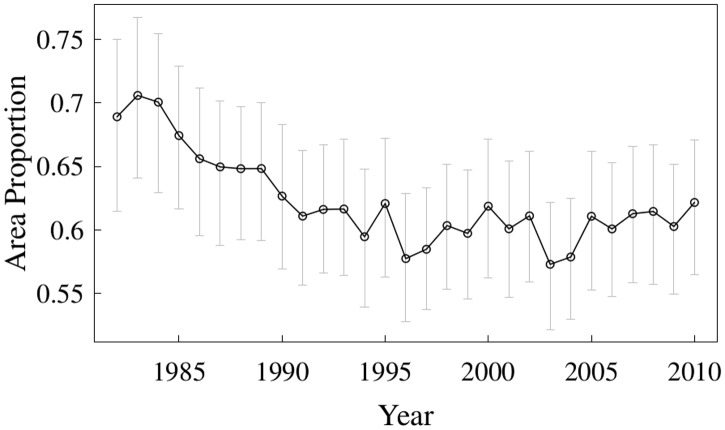
Habitat occupancy index. Minimum area containing 95% of the estimated cod abundance by year.

We calculated a”habitat occupancy index” as the minimum area containing 95% of the estimated abundance by year. The results showed that when cod population size was high, the occupancy index was above 70%, while when the population decreased the habitat occupied was reduced to 57% (Fig 5). After the 2005, a slight increase in the area occupied has occurred.

## Discussion

Our study shows, using for the first time a long time-series of bottom trawl survey data, the large changes in spatial distribution experienced by the Eastern Baltic cod during the past three decades. Population densities were high in large areas of the central Baltic in the first years of our time series up to mid-1980s, whereas thereafter the population has shown a progressive contraction into the southwest area (i.e. SD 25). Our statistical analyses suggest that during the past three decades the geographical distribution of the Baltic cod population has been driven by both density-dependent processes and hydrographic conditions, and intraspecific interactions played a more relevant role.

We found that a model containing the spatially variant density-dependent term, where the effect of cod population size varies over the Baltic landscape, was able to best capture the spatio-temporal dynamics of the Baltic cod, and improve the predictability of the model. This model revealed that cod density in the northeastern area decreased more than in the rest of the Baltic Sea during the rapid decrease of the population size in the 1980s, resulting in a spatial contraction of the cod population after the late 1980s into the southwest area. This was pictured by the time-series of area occupied in SDs 25–28, which was reduced of approximately 20%. This spatial contraction may have been facilitated by a decrease in salinity in the northern areas [31], with cod therefore likely leaving these areas when the salinity had become too low. However, the salinity in SD 28 increased again after the late 1980s, but without a re-expansion of the cod population to the north, likely because of the lasting low population size. After 2005, the oxygen level at the main average depth of cod distribution has always been higher in the northern areas and therefore hardly triggering the contraction of cod population into the south. Our results also revealed a minor positive effect of the regional (i.e. within a SD) reproductive volume on the local cod densities three years later, supporting the idea that individuals born in a SD tend to remain in that area. Accordingly, the contraction of the cod population into southwest areas (i.e. SD 25) after the early 1980s could have been also related to the loss of suitable spawning habitats (in terms of water masses with oxygen and salinity appropriate for spawning and ensuring egg survival) in the northern SDs [58]. The partial effects of our model also revealed that cod density was the highest at oxygen concentrations of 3–6 ml/l and salinity levels of 10–12 psu, indicating these as the preferred hydrological conditions for adult cod at the local scale.

We found that spatial density-dependence was more important to explain local cod density than the regional reproductive volume (acting on recruitment) and the local hydrological conditions (acting on habitat choice). As density increases in a habitat, per-capita resources become scarce, and individuals are expected to move towards marginal, less suitable habitats, but favored by lower local competition [59]. Different theoretical models have been proposed to describe changes in species density and distribution in relation to variations in their global abundance [25, 60], and a large variety of density-dependent spatial dynamics has been supported by empirical data on several marine and terrestrial species (e.g. see [19, 61–63]). Variability in the density-dependent responses have been found among different fish populations, but also within the same population under different conditions [14], and throughout the ontogeny [22]. The quality and richness of the data we analysed, and the wide range of environmental conditions, and population size experienced by the Eastern Baltic cod, during the time period investigated, allowed to disentangle the effect of the major drivers of its spatio-temporal dynamics, and suggested that density-dependent habitat selection may play a major role for this population.

However, a positive relationship between population size and distribution does not prove *per se* density-dependent habitat selection because the relationship could be due to a common factor driving both population abundance and area occupied [23]. For the Baltic Sea cod, for example, hydrological variations (e.g. in salinity and oxygen) influence both recruitment [45] and spatial distribution (our study). However, in presence of density-dependent habitat selection, the fish distribution should result in average fitness being equal between habitats [64]. In marine fishes, proxies for growth, such as size-at-age or condition, are life-history traits commonly considered good indicators of the individual reproductive success and thus fitness [65, 66]. Therefore, spatial variation in size-at-age or body condition should be a reasonable test of the theory [23]. In the case of the Baltic cod, the individual condition has shown large variations during the past thirty years, but these variations were very similar in all the areas of the central Baltic Sea (S4 Fig). Moreover, during the late 1970s-early 1980s, at the highest level of cod population size, the cod mean size-at-age was low [37], whereas after the stock crashed size-at-age increased, indicating density-dependent growth due to high competition among cod individuals. Under these circumstances, cod might tend to search food over a wider area in the Baltic landscape and therefore expand its spatial distribution. As an evidence of this expectation, during the late 1970s and early 1980s, when the cod population size peaked, the cod population was distributed in areas usually not occupied by this species, as the Gulf of Riga (SD 28.1) where cod is not able to recruit due to unfavorable hydrological conditions [29]. In this period, high commercial landings of cod were also reported from other marginal areas, such as the Bothnian Sea and Gulf of Finland (ICES official annual reports, Fig 2), suggesting a cod expansion into wider areas of the Baltic landscape at high population sizes. Our model predicted high CPUEs in the northern areas of the Central Baltic (SDs 27 and 28) during the early 1980s proving the capability to pick the expansion of the population distribution into northern areas in this period. On the other hand, at low population sizes, the individuals concentrate in the preferred more marine environment encountered in the south-western areas of the Baltic (SD 25).

**Fig 6:**
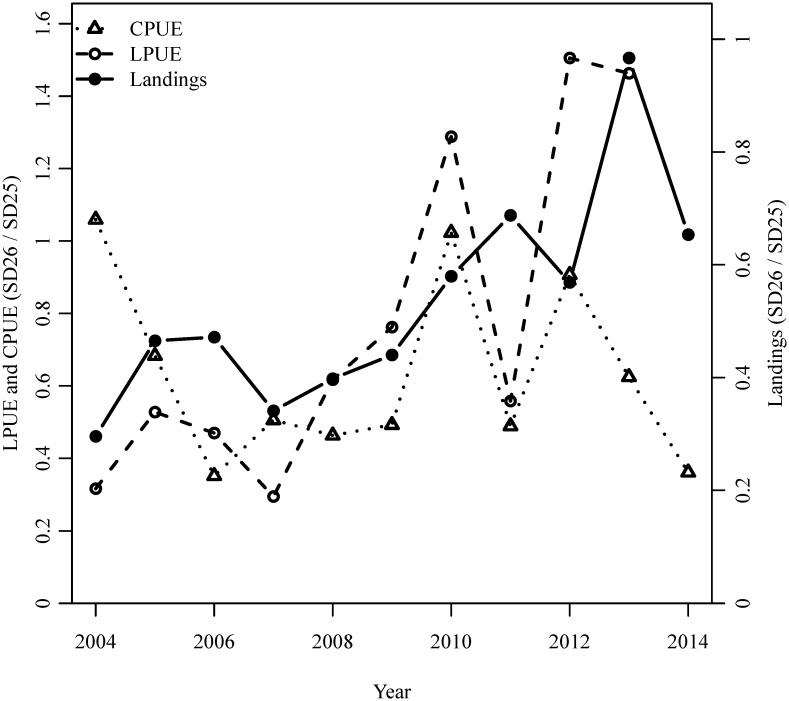
Indicator of the recent spatial development of the cod population. Time series of cod commercial landings (proportion of landings in SDs 25–26 caught in SD 26; data from ICES stock assessment working group reports), cod commercial landings per unit of effort (LPUE in kg landed/hour, ratio SD 26 / SD 25; data from the Scientific, Technical and Economic Committee for Fisheries of the European Commission) and survey catch per unit of effort (CPUE in kg/h, ratio SD 26 / SD 25; data from ICES DATRAS database).

Since 2007 the cod population has shown a slight recovery from the very low abundances estimated during the period 1990–2006 [67]. Therefore, according to our model an expansion of the distribution should have occurred after 2006. Data from commercial fisheries (both in terms of landings and landings-per-unit-effort) show a sharp proportional increase in cod landings in SD 26 with respect to SD 25 after 2005, suggesting an eastward expansion of the population (Fig 6). The expansion seems however not to have stretched into more northern areas (e.g. SDs 27 and 28). Currently we can only speculate about these observations, even if the lack of strong expansion northwards after the 2005 could be due to 1) the greatly increased extent of hypoxic and anoxic bottoms in northern SDs [68] and/or 2) the currently very low body condition of cod individuals potentially reducing the energy available for extensive movements, as shown for example for herring [69] and according to bioenergetic models for Atlantic cod [70]. Moreover, one part of the relation between population size and spatial distribution revealed by our model could be explained by movements in the offshore-coast direction (i.e. also within the same SD) and not necessarily only over larger north-south or east-west spatial scales across SDs [32]. This could explain the fact that the Baltic International Trawl Survey (BITS), not covering the most coastal areas (the survey covers mainly the areas deeper than 20 m), does not reveal trends in the proportion of cod caught between SDs as clear as the commercial catches (Fig 6). Moreover, we cannot exclude that the lack of a recent expansion into northern areas is due to the loss of spawning sub-components own to the intensive and persisting fishing occurred in the 1980s and 1990s [46].

In our analyses we did not include potentially important factors affecting cod distribution, such as prey distribution and fishing pressure. However, Casini et al. [31] showed that sprat density has increased in the northern Baltic Sea during the past twenty years, whereas it decreased in the southern areas. Herring has also shown an increased abundance in the northern areas of the central Baltic Sea during the study period [58]. These observations suggest that the contraction of the cod population in the southern Baltic since the early 1980s cannot be explained by changes in the pelagic prey distribution. Long-term spatially-resolved data on fishing pressure are not currently available and could not be included in our analyses, but if available in the future they should be taken into account in further analyses on the Baltic cod spatial distribution to understand the fishery effect on cod at different spatial scales [13].

Our results support previous findings that the spatiotemporal dynamics of fish populations are better explained when both density-dependent and density-independent processes are simultaneously accounted for [17, 18]. Our study illustrates also the importance of considering the spatial dimension in the analysis of fish populations dynamic because of the strong spatial component in the interplay between intraspecific interactions and environmental forcing. In a recent study, [29] provided evidence that the presence of cod in the northern areas of the Central Baltic produced a cod spillover into the adjacent Gulf of Riga (Fig 1) with direct and indirect top-down effects on all the trophic levels of the local ecosystem. Also in the Bothnian Sea the occasional presence of cod when the population peaked has been suggested to trigger a top-down control of the local commercially exploited herring population [71]. Similarly, the decrease of cod in coastal areas may have contributed to the recent increases in three-spined stickleback observed in the Baltic Sea, with vast secondary effects on coastal food webs [32, 72]. Therefore understanding and predicting the changes in cod spatial distribution is of crucial importance for fisheries management and the biological conservation of the whole Baltic Sea meta-ecosystem. Studying the changes in spatial distribution and area occupied by species is also considered fundamental in biodiversity conservation, as underlined for example by the EU Marine Strategy Framework Directive which has identified species area occupancy as one of the key indicators of ecosystem state [27].

## Supporting information

S1 Fig Spatial distribution cod sampled abundance. Quinquennial maps of cod CPUE in the Baltic International trawl Survey (BITS) from 1982 to 2007. Bubble size is proportional to cod CPUE.
(PDF)

S2 Fig Spatial distribution of bottom oxygen. Quinquennial maps with the spatial distribution of bottom oxygen concentration from the Swedish Coastal and Ocean Biogeochemical model coupled to the Rossby Centre Ocean circulation model (RCO-SCOBI) from 1982 to 2007.
(PDF)

S3 Fig Spatial distribution of bottom salinity. Quinquennial maps with the spatial distribution of bottom salinity from the Swedish Coastal and Ocean Biogeochemical model coupled to the Rossby Centre Ocean circulation model (RCO-SCOBI) from 1982 to 2007.
(PDF)

S4 Fig Temporal development of cod condition in different Subdivisions (SDs) of the Baltic Sea. Time-series of cod Fulton’s condition factor (weight/length^3^) in our study area, sampled by Sweden during the BITS survey in February-March.
(DOCX)

S5 Fig Best model diagnostics. (a) frequency distribution of model residuals; (b) estimated variance-to-mean relationship (solid line, slope equals to 268). The circles are averaged squared residuals in each category (e.g. 0 < E(Y) < 2, 2 < E(Y) < 4 and so on); (c) semivariogram of the model residuals.
(PDF)

S1 Table Summary statistics of the full model.1 and reduced versions ‘leaving-out-one’ term at time. Intercept (A) and estimated degrees of freedom for each term of the full best model (model.1) and reduced models ‘leaving-out-one’ term at time. The deviance explained (dev.expl.) and the % change in the dev.expl. are reported for each reduced model in relation to the full best model (model.1).
(DOCX)

S1 Text Estimating conversion factors among trawl types by cross-testing abundance indices among successive survey time series. (DOCX)
