# Supplementary figures and images for: Spatio-temporal dynamics of a fish predator: Density-dependent and hydrographic effects on Baltic Sea cod population
Source: PLoS One. 2017 Feb 16;12(2):e0172004. doi: 10.1371/journal.pone.0172004 (PMC5313222; doi:10.1371/journal.pone.0172004)

**1982**

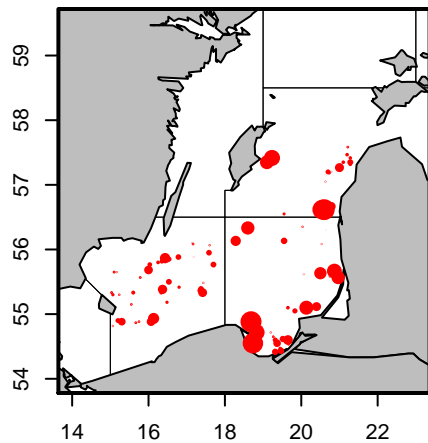

**1987**

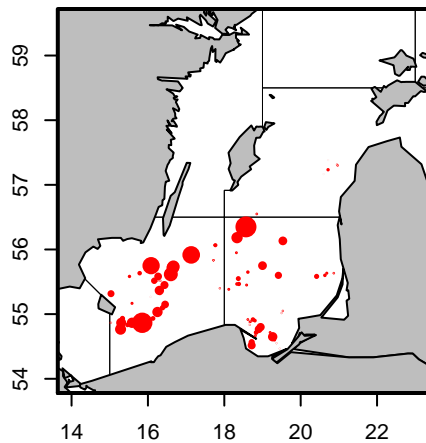

**1992**

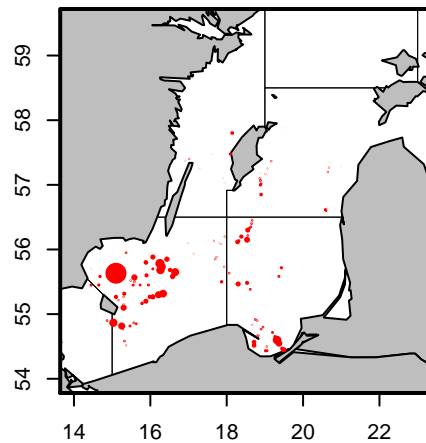

**1997**

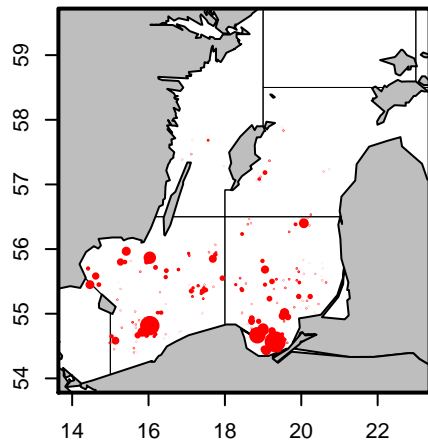

**2002**

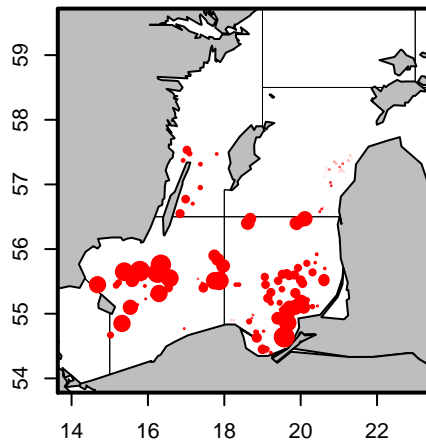

**2007**

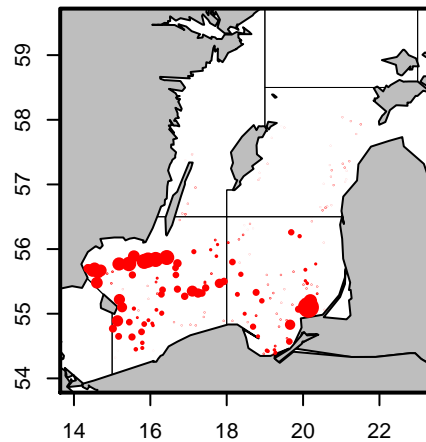

Latitude

Longitude

Supplement: S1 Fig — Quinquennial maps of cod CPUE in the Baltic International trawl Survey (BITS) from 1982 to 2007. Bubble size is proportional to cod CPUE. (PDF) [file pone.0172004.s001.pdf]

**1982**

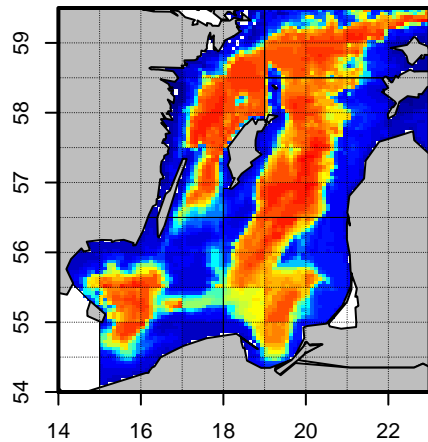

**1987**

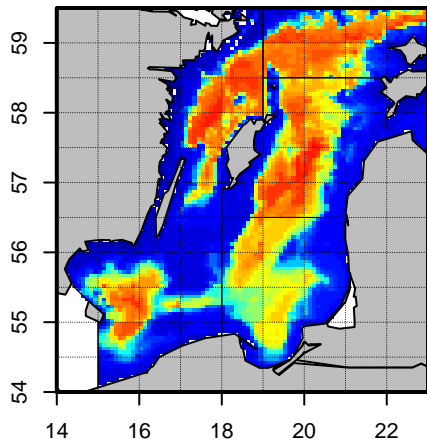

**1992**

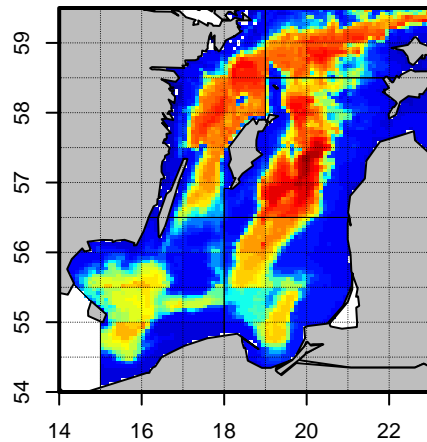

**1997**

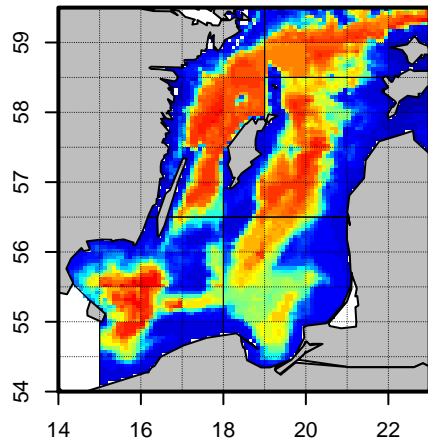

**2002**

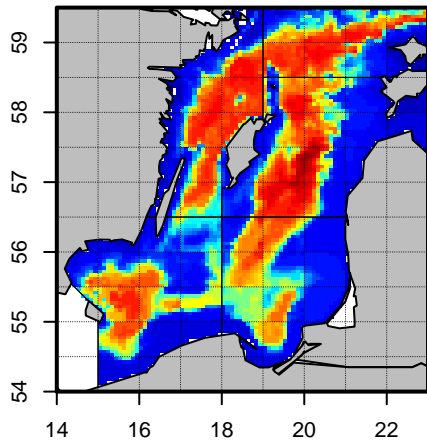

**2009**

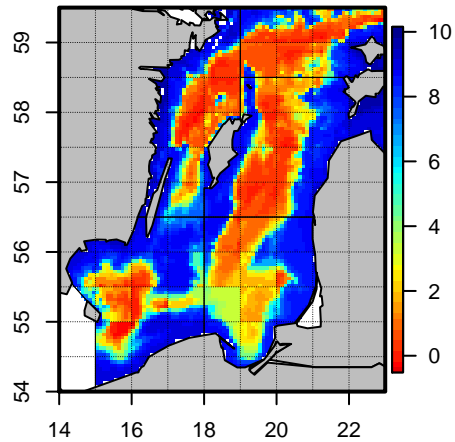

Latitude

Longitude

Supplement: S2 Fig — Quinquennial maps with the spatial distribution of bottom oxygen concentration from the Swedish Coastal and Ocean Biogeochemical model coupled to the Rossby Centre Ocean circulation model (RCO-SCOBI) from 1982 to 2007. (PDF) [file pone.0172004.s002.pdf]

**1982**

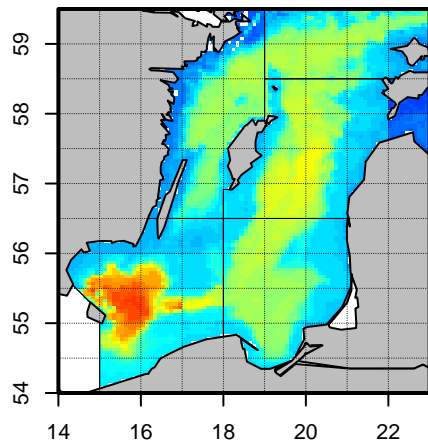

**1987**

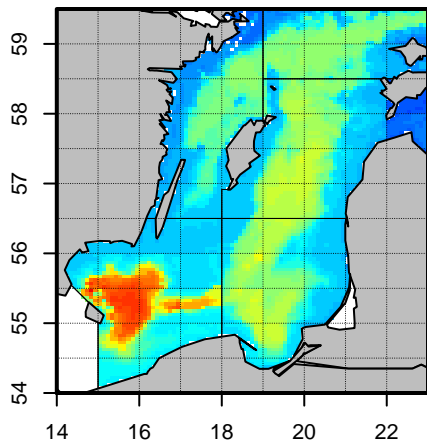

**1992**

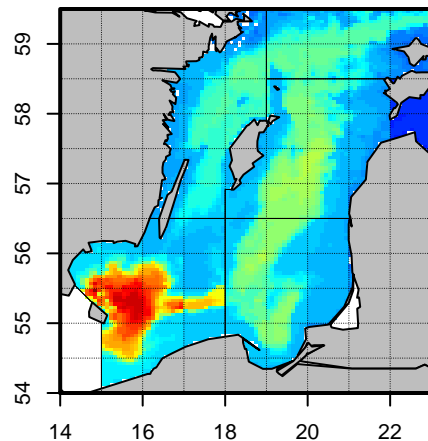

**1997**

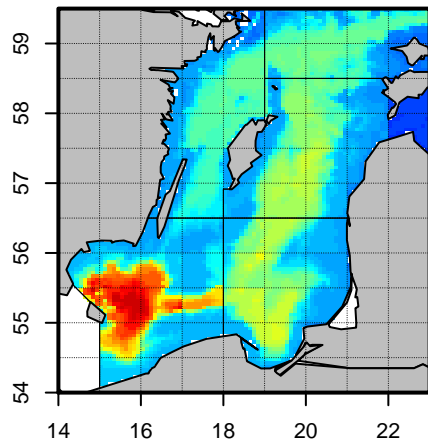

**2002**

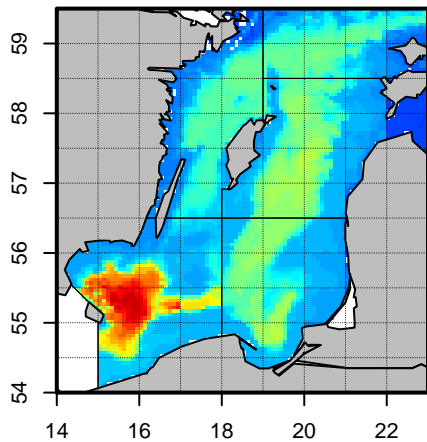

**2009**

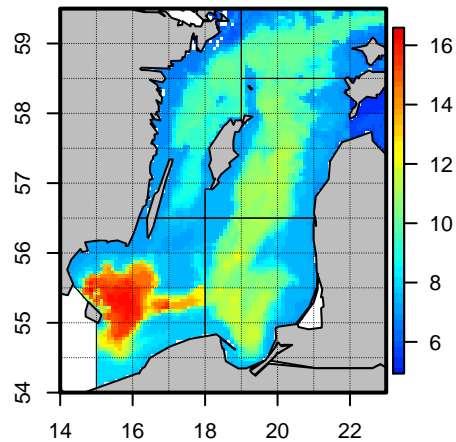

Latitude

Longitude

Supplement: S3 Fig — Quinquennial maps with the spatial distribution of bottom salinity from the Swedish Coastal and Ocean Biogeochemical model coupled to the Rossby Centre Ocean circulation model (RCO-SCOBI) from 1982 to 2007. (PDF) [file pone.0172004.s003.pdf]

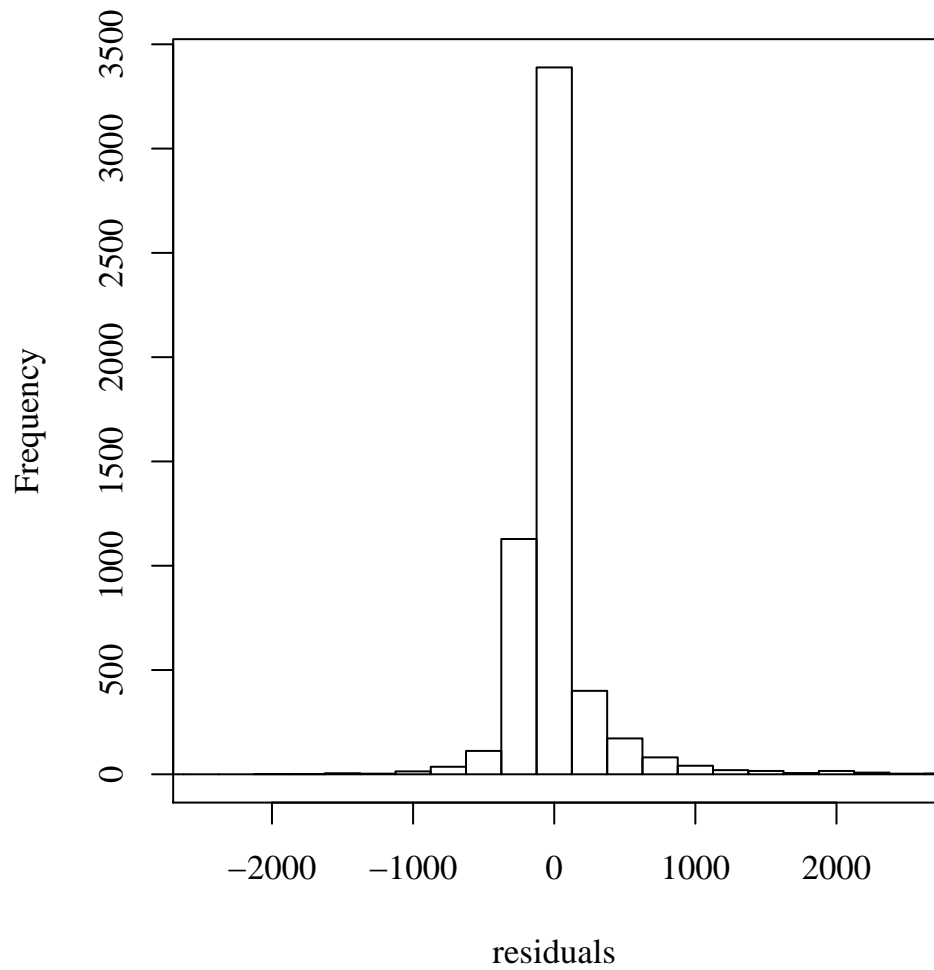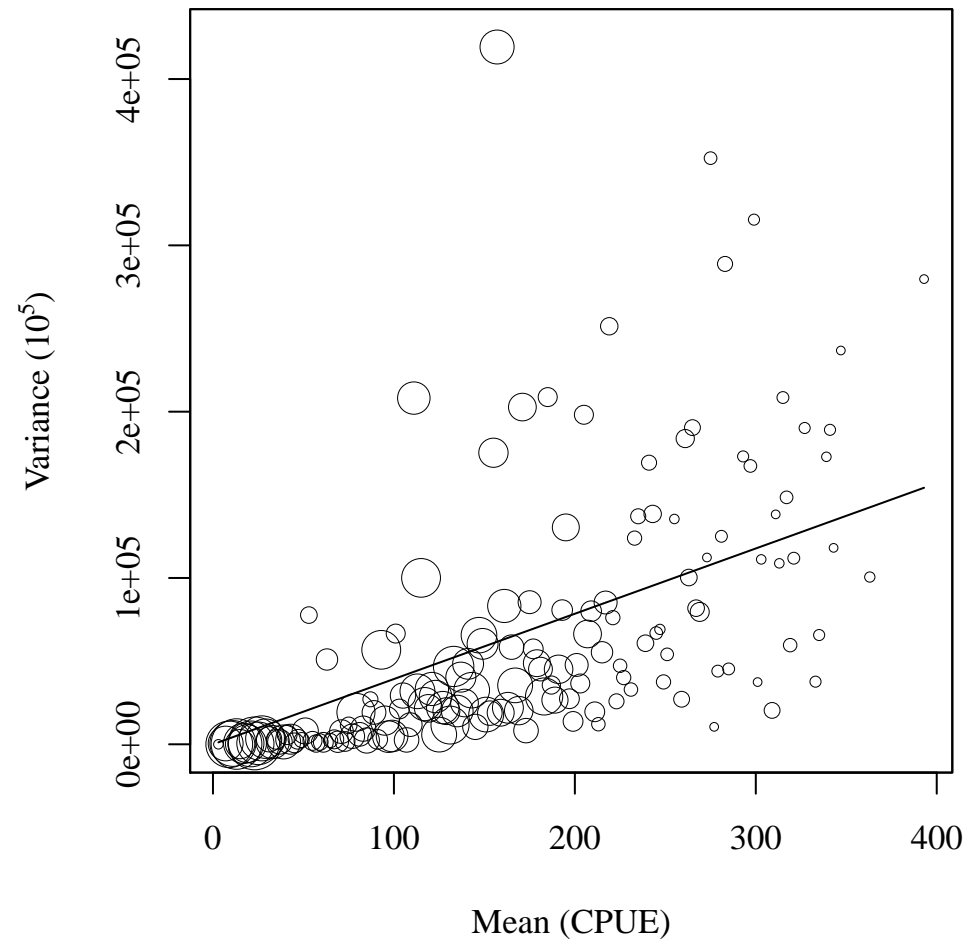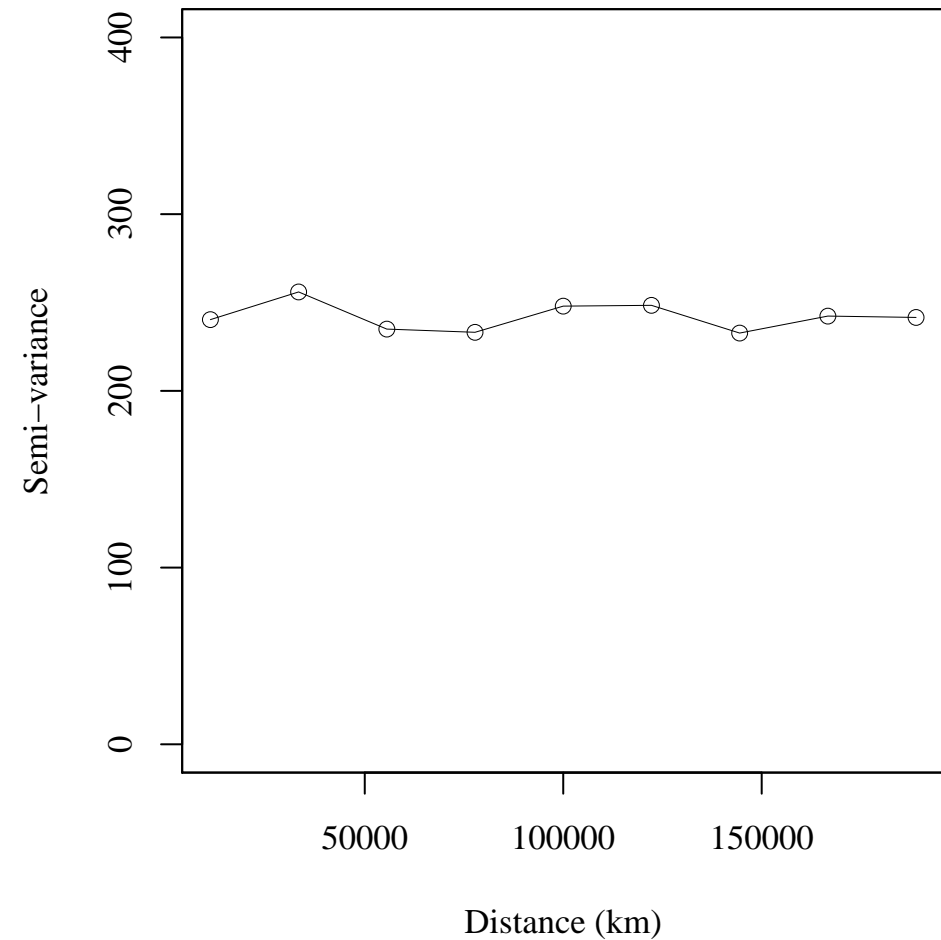

Supplement: S5 Fig — (a) frequency distribution of model residuals; (b) estimated variance-to-mean relationship (solid line, slope equals to 268). The circles are averaged squared residuals in each category (e.g. 0 < E(Y) < 2, 2 < E(Y) < 4 and so on); (c) semivariogram of the model residuals. (PDF) [file pone.0172004.s005.pdf]
