# Supplementary material for: Spatio-temporal dynamics of a fish predator: Density-dependent and hydrographic effects on Baltic Sea cod population
Source: PLoS One. 2017 Feb 16;12(2):e0172004. doi: 10.1371/journal.pone.0172004 (PMC5313222; doi:10.1371/journal.pone.0172004)
Supplement: S4 Fig — Time-series of cod Fulton’s condition factor (weight/length3) in our study area, sampled by Sweden during the BITS survey in February-March. (DOCX) [file pone.0172004.s004.docx]

**Spatio-temporal dynamics of a fish predator: density-dependent and hydrographic effects on Baltic Sea cod population**

Valerio Bartolino^1^, Huidong Tian^1^, Ulf Bergström^2^, Pekka Jounela^3^, Eero Aro^4^, Christian Dieterich^5^, Markus Meier^5,6^, Massimiliano Cardinale^1^, Barbara Bland^1^ and Michele Casini^1*^

**Supporting information**

**S6 Figure. Temporal development of cod condition in different Subdivisions (SDs) of the** **Baltic Sea.** Time-series of cod Fulton’s condition factor (weight/length^3^) in our study area, sampled by Sweden during the BITS survey in February-March.
