# Supplementary material for: Spatio-temporal dynamics of a fish predator: Density-dependent and hydrographic effects on Baltic Sea cod population
Source: PLoS One. 2017 Feb 16;12(2):e0172004. doi: 10.1371/journal.pone.0172004 (PMC5313222; doi:10.1371/journal.pone.0172004)
Supplement: S1 Table — Intercept (A) and estimated degrees of freedom for each term of the full best model (model.1) and reduced models ‘leaving-out-one’ term at time. The deviance explained (dev.expl.) and the % change in the dev.expl. are reported for each reduced model in relation to the full best model (model.1). (DOCX) [file pone.0172004.s006.docx]

|  |  |  |  |  |  |  |  |  | |  | |  | |
| --- | --- | --- | --- | --- | --- | --- | --- | --- | --- | --- | --- | --- | --- |
| Model |  | Predictors | | | | | | |  | | dev.expl | | % change dev.expl |
|  |  | A | s(lon,lat) | s(lon, lat)· population | s(oxy, sal) | s(depth) | s(RV) |  | |  | |  | |
| full model.1 | | 4.2 | 26.1 | 18.4 | 21.9 | 6.3 | 8.5 |  | | 44.0 | |  | |
|  |  | 4.4 |  | 28.1 | 22.8 | 6.3 | 8.6 |  | | 40.3 | | -8.4 | |
|  |  | 5.0 | 28.0 |  | 26.8 | 6.4 | 8.5 |  | | 30.1 | | -31.7 | |
|  |  | 4.2 | 26.1 | 21.2 |  | 6.2 | 8.5 |  | | 41.7 | | -5.3 | |
|  |  | 4.3 | 26.1 | 16.6 | 24.2 |  | 8.4 |  | | 40.8 | | -7.4 | |
|  |  | 4.3 | 26.1 | 18.6 | 21.3 | 6.3 |  |  | | 42.7 | | -2.9 | |

**S5 Table. Summary statistics of the full model.1 and reduced versions ‘leaving-out-one’ term at time.** Intercept (A) and estimated degrees of freedom for each term of the full best model (model.1) and reduced models ‘leaving-out-one’ term at time. The deviance explained (dev.expl.) and the % change in the dev.expl. are reported for each reduced model in relation to the full best model (model.1).
