# Supplementary material for: Spatio-temporal dynamics of a fish predator: Density-dependent and hydrographic effects on Baltic Sea cod population
Source: PLoS One. 2017 Feb 16;12(2):e0172004. doi: 10.1371/journal.pone.0172004 (PMC5313222; doi:10.1371/journal.pone.0172004)
Supplement: S1 Text — (DOCX) [file pone.0172004.s007.docx]

**Appendix S2**

**Estimating conversion factors among trawl types by cross-testing abundance indices among successive survey time series**

The Baltic cod stock has been monitored annually since 1982 through bottom trawl surveys. The national research vessels carried out by most countries surrounding the Baltic Sea used different gears and surveyed part of the area with some overlap in coverage.

In order to standardize the surveys, ICES established a Study Group on Young Fish Surveys in the Baltic in 1985. Different gears and survey designs were tested. However, agreement on a standard survey trawl type was not made and hence, different gear types were used throughout 1980s and 1990s. Only after agreement in 2000, a common standard TV-3 trawl (Nielsen et al. 2001) and a standard depth stratified sampling design were implemented resulting in the coverage of the whole Baltic Sea in the BITS survey.

The main aim of inter-calibration tests and gear change in 2000 was to enable a shift of the recruitment index used in stock assessments of Baltic cod from an age of 2 years to 1 year. This was achieved by an introduction of a new TV-3 trawl that had better selectivity for the age group 1 relative to the traditional national trawls. Also the survey design was modified to more extensively cover age group 1 in order to get more reliable forecasts of all age groups.

Comparative hauls were made to inter-calibrate catching efficiency of traditional national trawls used from before 2000 with the new standardized TV-3 trawl used from 2000. The work with standardizing gear and creating conversion factors was done mainly under the EU project ISDBITS (Nielsen et al. 2001). These inter-calibration tests primarily regarded cod catch per unit of effort (CPUE; Nielsen et al. 2001; Oeberst and Grygiel 2002, 2004).

The used alternate haul method, however, produced variable conversion factors. Thus, work on the inter-calibration between the old and new trawl types continued in 2001-2003 under the auspices of the ICES (Baltic International Fish Survey Working Group; Anon. 2001). Their analyses showed that first tow to some extent influenced the result of the following tow and that the sequence of the gears influenced the results of the hauls (Anon. 2001). Moreover, Grygiel (2004) found that, using the same TV-3 survey trawl, the average CPUE of cod was 39% higher in first hauls than in second (repeated) hauls. Also Levy et al (2004) found that the disturbance effect of the TV3 trawl was quite significant, being estimated at about 0.4, implying that the fish density available for the subsequent haul was reduced by 60%. These findings suggested that the conversion factors obtained by alternating gears would be severely influenced. Consequently, errors and unexplained variability in survey indices impacted estimates of recruitment.

The method applied aims to estimate conversion factors by cross-testing trawls’ catching efficiencies at all sites among successive survey time series. Temporal cross-tests were computed with an array of computational intelligence techniques, in particular neural network (NN) modeling. Our main goal was to obtain reliable conversion factors among old and new trawl types. Reliable conversion factor estimates are fundamental because CPUE is a main piece of information in the evaluation of recruitment.

**Method**

The gear conversion approach used here resembles that derived for the Pacific yellowfin tuna longline fishery (Hinton and Maunder 2004). However, the approach presented here tested gear performances among two successive survey time series, whereas in the assessment of Pacific yellowfin tuna longline fishery gear performances were tested within a time series. This is because in the present study catching efficiencies of the trawls were in different standards and the trawls were used in different times, whereas in the Pacific yellowfin tuna longline fishery un-standardized gears were used concurrently.

The methodology used here also resembles those used in forecasting (see Tashman 2000; Fildes and Makridakis 1995). The difference was that we parameterized the model backwards in time with known (used) testing data, whereas in forecasting the model performances (prediction powers) are tested forwards in a time series with unknown (unused) data.

The methodology is divided into three sections: (1) preprocessing of data, (2) training and testing the performance of a model and, (3) selection of the best performing model on the basis of testing performance (FACTS project, <http://www.facts-project.eu>). The mathematical methods to estimate conversion factors in this study employed an array of neural network (NN) modeling (in some cases also referred to as “machine learning”, “ML” or “computational intelligence”, “CI”) techniques.

**Data**

The CPUE samples of cod were collected under the Baltic International Trawl Survey (BITS) in the Baltic Sea Main Basin (SDs 25 – 28). The CPUE samples from 1982 – 1999 were collected using traditional national trawl types and, the samples from 2000 – 2009 were collected using new standardized TV-3 trawl type.

The present study used gear standardized BITS CPUE data. The total number of test hauls in the BITS database was 5420 that each consisted of 10 cod age groups (AGs 1 – 10). The model used here included 19930 samples (observations, rows) from years 2000 – 2009 and, 5340 samples from years 1998 – 1999 i.e. the BITS trawl CPUE time series from before 1998 were not used in the model. The proportion of testing data was 21% of all data. In general, a proportion of samples used for testing purposes in other NN studies vary between 10% - 50%.

**Preprocessing**

The independent variables were transformed to equalize the effect variables have on model output. The categorical independent variables (age group, year, country) were zero-one coded (one for each possible category) and, the continuous independent variables (latitude, longitude, depth and month) were standardized (zeroed mean and variance of one). The dependent variable (CPUE) was log-transformed (ln + 1) to improve fit and to equalize variances.

**Training and testing**

NNs learn dependencies from data and to improve the generalization performance of the NNs, the input data are in most cases divided into 2-3 subsets: a training set, a testing set and/or a validation set. By “generalization” we refer to a model producing reasonable outputs on the basis of testing set not encountered during training. In the present study, the standard TV-3 CPUE data from years 2000 – 2009 were used (tagged) as a training set, whereas the standardized national trawl CPUE data from years 1998 – 1999 were tagged as a testing set. The categorical year effect of a testing set was tagged as if the testing set would (randomly) overlap with years 2000-2001. By inserting synthetic observations we reconstructed an even sampled time series. Consequently, the assumption included here was that the abundance of cod was approximately leveled among years 1989-2001. The remaining factors i.e. 6 independent variables and the dependent variable were treated “as is”. Figure 1 shows the two time series and their parameterization in the model using training - testing method.

**Model selection**

The training - testing trials were run (repeated) until the improvement of the testing error (mean squared error, MSE) was lower than 1% during the last one hour of parameter optimization trials. Then, the trained model that tested the best was selected. The relationships between the actual and predicted CPUE of the training set were the trawl conversion factors in 2000 – 2009. The resulting model converted catching efficiency of a TV-3 trawl to that of the traditional trawls.

**Model**

A neural network (NN, see Bishop (1995); specifically generalized regression neural network, GRNN, Specht (1991)) was used to estimate conversion factors among traditional and new trawl types. In general, NN models allow the data to determine the relationships among variables instead of the researcher imposing some specific relationships or assumptions of the response variable. The advantage is that, in contrast with parametric regression models, a GRNN does not assume some a priori selected functional relationship when recognizing CPUE patterns at all sites. Specht (1991) describes the algorithm in full detail. The general use of NNs in fisheries research is described by Suryanarayana et al. (2008) and that in ecology by Lek and Guégan (1999).

**Results**

In total, 124 training – testing trials were required to minimize MSE of a testing set (Table 1). The root mean square error (RMSE) of the training set was 0.31, which is 0.37 fish (N) per unit of effort, on average . The RMSE of the testing set was 1.44, which is 3.23 fish on average. Obtaining smaller RMSE in the training set than that in the testing set is normal with large datasets. With small databases in some cases, a smaller testing than training error may occur by chance.

The coefficient of variation (R2) in the training set was 0.97 and, that in the testing set it was 0.41 (Figure 2). The correlation coefficient between the actual and predicted CPUE in the training set was 0.99 and, that in the testing set it was 0.64.

The residual distributions in the training set did not show any clear pattern (Figure 3a,b). i.e. the trained NN model was able to follow the actual TV-3 trawl CPUE patterns during 2000 – 2009. The residuals in the testing set, however, were more spread than those in the training set (Figure 3c,d). That is because the spatial distribution of fish and test hauls was different in 1998 – 1999, than in 2000 – 2001.

The reader is reminded that the predicted CPUE level of TV-3 trawl refers to the CPUE levels (catching efficiency) of traditional national trawls. That is because the training set was parameterized towards the probability distribution of a testing set.

The average back-transformed conversion factor i.e. the back-transformed multiplier between the actual and predicted CPUE of TV-3 trawl over all training examples was 1.44 (49.61Actual / 34.42Pred). That is, the back-transformed average predicted CPUE values of a TV-3 trawl were 69% (34.42Pred / 49.61Actual) of the actual ones in 2000 – 2009. The difference between the predicted and actual catching power of a TV-3 trawl was highest in age groups 1 – 3 (Figure 4). That is, the TV-3 trawl more effectively caught (selected) cod age groups 1 – 3, than the traditional trawls. In cod age groups 4 – 10, the differences in back-transformed catching powers between traditional and new trawl types were smaller.

**Conclusions**

Predicted CPUE levels correlated well with actual ones in the southern Baltic Sea. The catching efficiency of a standard TV-3 trawl was parameterized towards catching efficiency of traditional national trawls. Catching efficiencies within and over the two survey data sets were predictable and hence, the algorithm was able to capture conversion factors of survey trawls at all sites when they changed over time.

ICES (Anon. 2001) analyzed alternate haul based log-transformed inter-calibration data with a general linear model (GLM). Their average in-sample error (RMSE) between predicted catching efficiencies of new TV-3 trawl versus traditional national trawls was 1.55 (Anon. 2001). Here the testing error was somewhat smaller (1.44), i.e. NN based testing method was able to recognize catching efficiency patterns of the traditional national trawls. Further, a very small in-sample training error (0.31) proofed NN model’s ability to recognize catching efficiency patterns of the TV-3 trawl during 2000 – 2009. The statistical performance of the model suggests that the use of comparative test haul data is not a necessity when estimating conversion factors among survey trawls.

Lewy et al. (2004) derived a method to inter-calibrate catching efficiencies among survey trawl types independent of spatial fish distribution. They found that the new TV-3 trawl was significantly more efficient than the Danish Granton trawl, especially for cod less than 20 cm. This finding is roughly in line with our results, as the TV-3 trawl more effectively selected age groups 1 – 3 than the traditional national trawls. This finding also supported a priori expectations, as the new trawl was larger both vertically and horizontally and used a rubber snake ground gear instead of the bobbin arrangement used earlier (Lewy et al. 2004).

Oeberst and Grygiel (2004) estimated that the mean conversion factors for cod larger than 24 cm were 1.8 and 1.13 for the Polish and German experiments, respectively. In the present study, similar comparisons between the national gears would not have been relevant because the catching efficiencies of national gears (testing data) had already been standardized by ICES. However, the mean conversion factor across all samples in our study was roughly at the same level (1.4) as that in the study of Oeberst and Grygiel (2004).

We computed temporal cross-tests among gear performances at all sites and assumed leveled abundance of cod between periods 1998 – 1999 and 2000 – 2001. Earlier trawl conversion studies have mostly assumed leveled abundance of fish between accurately positioned first and second hauls i.e. immobility of fish (and gear) within some time interval. When analyzing catching efficiencies within the same track line, however, it may be impossible to distinguish between the disturbance net effects that include both the removal of fish caught by the first haul and induced behavioral effects that influence migration of fish in the neighborhood of the trawl track line (Levy et al. 2004). In order to overcome this problem, some gear conversion studies have compared (or averaged) gear performances between nearby haul track lines. This survey setup, however, could result influenced conversion factor estimates due to (possibly) uneven density of fish between the nearby track lines. Clearly, different survey setups and assumptions should be cross-tested and validated in a closed system in order to derive scientific conclusions about their absolute superiority.

The statistical performance of our approach was either comparable, better, or far better than that in the earlier studies. This suggests that the presented approach could be a potential alternative to the more traditional conversion factor studies. Given the enormous amount of resources and costs to conduct inter-calibration test surveys, our approach is certainly arguable. These survey indices may be the only source of information on which to base management advice and hence, greater precision and accuracy as well as lower costs in estimating conversion factors are highly desirable.

**References**

Anon. 2001.. Report of the Baltic International Fish Survey Working Group. Kaliningrad, Russia 5–9 February 2001. ICES CM 2001/H:02, Ref.: D, 252 pp.

Bishop CM. 1995. Neural networks for pattern recognition. Oxford: Oxford University Press.

Fildes, R. and Makridakis, S. 1995. The impact of empirical accuracy studies on time series analysis and forecasting. International Statistical Review, 63, 289–308.

Hinton, M.G., Maunder, M.N. 2004. Methods for standardizing CPUE and how to select among them, Collective volume of scientific papers. International Commission for the Conservation of Atlantic Tunas/Recueil de documents scientifiques Commission internationale pour la Conservation des Thonides de l’Atlantique/Coleccion de documentos cientificos. Comision, internacional para la Conservacion del Atun Atlantico [Collect. Vol. Sci. Pap. ICCAT/Recl. Doc. Sci. CICTA/Colecc. Doc. Cient. CICAA], 2004;56(1),169–177.

Lek S, Guégan JF. 1999. Artificial neural networks as a tool in ecological modelling, an introduction. Ecological Modelling, 120:65–73.

Lewy, P. Nielsen, J.R. Hovgård, H. 2004. Survey gear calibration independent of spatial fish distribution. Canadian Journal of Fisheries and Aquatic Sciences, Volume 61, Number 4, pp. 636-647(12).

Oeberst, R. and Grygiel, W. 2002. Analyses of conversion factors. Working paper [in:] Report of the Baltic International Fish Survey Working Group ICES CM 2002/G:05, Ref. H; 108-118.

Oeberst, R. and Grygiel, W. 2004. Estimates of the fishing power of bottom trawls applied in the Baltic fish surveys. Bulletin of the Sea Fisheries Institute, Gdynia, 1(161): 29-41.

Olden JD, Jackson DA. Illuminating the ‘‘black box’’: a randomization approach for understanding variable contributions in artificial neural networks. Ecological Modelling 2002;154:135–50.

Specht DF. 1991. A generalized regression neural network. IEEE Transactions on Neural Networks, 2:568–76.

Suryanarayana, I, Braibanti, A, Rao, R.S., Ramam, V.A., Sudarsan, D., Rao, G.N. 2008. Neural networks in fisheries research. Fisheries Research, 92:115–39.

Tashman, L.J. 2000. Out-of-sample tests of forecasting accuracy: an analysis and review. International Journal of Forecasting, 16:437–50.

**Tables**

Table 1. The summary statistics of the GRNN model.

| ***Configuration*** |  |
| --- | --- |
| Independent Category  Variables | Country, year,  age group |
| Independent Numeric  Variables | Latitude, longitude, depth, month |
| Dependent Variable | LN(CPUE+1) |
| ***Training*** |  |
| Number of Cases | 19930 |
| Training Time (h:min:sec) | 4:39:18 |
| Number of Trials | 124 |
| Root Mean Square Error | 0.3173 |
| Mean Absolute Error | 0.1708 |
| Std. Deviation of Abs.  Error | 0.2674 |
| ***Testing*** |  |
| Number of Cases | 5340 |
| Root Mean Square Error | 1.4430 |
| Mean Absolute Error | 0.9306 |
| Std. Deviation of Abs.  Error | 1.1030 |

**Figures**

**Figure 1.** The two standardized BITS CPUE data sets (a) and, tagged training + testing windows in the model (b). The categorical year effect of the testing set was tagged as if it would overlap randomly with years 2000 – 2001. The remaining factors (6 independent variables and the dependent variable) were treated “as is”. In the training – testing trials, the training set was parameterized towards the probability distribution of a testing set. Consequently, the relationships between the actual and predicted training samples were the conversion factors among trawl types in years 2000 – 2009.

Figure 2. Predicted vs. actual CPUE in the training set (a) and in the teing set (b).

**Figure 3.** Residual distributions of the training set (a,b) and the testing set (c,d).

**Figure 4.** Back-transformed conversion factors by age groups 1 – 10 in years 2000 – 2009. The words “predicted” and “actual” refer to the catching efficiencies of the traditional national trawls and the TV-3 trawl, respectively. That is because the catching efficiency of the TV-3 trawl was parameterized towards the catching efficiency of the traditional national trawls.
